# Editorial: T Cell Metabolism in Infection

**DOI:** 10.3389/fimmu.2022.958276

**Published:** 2022-07-04

**Authors:** Duojiao Wu, Jin-Fu Xu

**Affiliations:** ^1^ Shanghai Key Laboratory of Lung Inflammation and Injury, Shanghai, China; ^2^ Institute of Clinical Science, Zhongshan Hospital, Fudan University, Shanghai, China; ^3^ Department of Respiratory and Critical Care Medicine, Shanghai Pulmonary Hospital, School of Medicine, Tongji University, Shanghai, China; ^4^ Institute of Respiratory Medicine, School of Medicine, Tongji University, Shanghai, China

**Keywords:** T cell, Metabolism, Infection, Immune function, pathogen

## Introduction

T cell is an essential component of the adaptive immune response in infection. Metabolic reprogramming on both the cellular and organismal levels of T cells is critical for eradicating infectious pathogens (1). With the growing concerns during the COVID-19 pandemic it requires further understanding of alterations in these metabolic processes for developing measures against such infection outbreaks. Through this collection of 5 articles, this special issue serves as a professional overview of recent progress in this field.

We feature two articles that describe the metabolic adaption of T cells in the context of infectious diseases. By using severe acute respiratory syndrome coronavirus 2(SARS-CoV-2), mycobacterium tuberculosis (MT) and human immunodeficiency virus infection (HIV) as examples, Wik and Skålhegg pinpointed that personalized metabolic intervention is required to aid pathogen clearance or confer resistance to infections. Generally, as reported before (2), activated cells need glycolysis to mount a robust response including rapid proliferation and synthesis of biomolecules (cytokine, antimicrobial proteins and lipid mediators). Actually, the process is more complicate. Triggered by the ligation of T cell receptors (TCR), T cell response to infection is a concerto, mingling mTORC1/mTORC2 adaptations ensuring adequate nutrient uptake and utilization to support the requirement of rapid growth and proliferation. The process is different between cytotoxic T cells (Tc) and helper T cells (Th). Quiescent Th cells rely mainly on oxidative phosphorylation (OXPHOS), the energy-preserving and catabolic dormancy programs to maintain homeostasis. While the effector subsets are characterized by increased the anabolic programs, including glycolysis, glutaminolysis, fatty acid synthesis and oxidation (FAO). Different viruses cause distinct effects on T cell metabolism. By inhibiting mTORC1 pathway of T cells, SARS-CoV-2 infection causes reduced glycolytic activity, increased mitochondrial dysfunction and apoptosis. In contrast, HIV induces enhanced OXPHOS to support viral replication.

However, in chronic viral infections, T cells exhibit impaired function with up-regulated expression of immunosuppressive molecules known as T-cell exhaustion (Tex). Zheng et al. summarized dysregulated metabolic profile of Tex driven by a combination of chronic antigenic stimulation and external multiple signals (nutrients, cytokines, certain microRNA, etc.). It is well accepted that the shifting from glycolysis to FAO for energy production is an important trigger of Tex development (3). However, there are some unresolved contradictions, such as fatty acids as potential metabolic fuels that help CD8^+^T cells maintain effector function in glucose-starved environments, meanwhile fatty acids also drive CD8^+^Tex development. Intensified FAO in mitochondria can cause mitochondrial depolarization, mitochondrial biosynthesis impairment and excessive reactive oxygen species (ROS) production, which are tightly associated with the dysfunction of Tex.

Mayberry et al. discussed that the metabolic regulation of T follicular helper (Tfh) in the context of autoimmune related disorders, cancer and infection. Several cytokine-related signalling pathways which are critical for Tfh development either facilitate or inhibit specific metabolic pathways of Tfh. BCL6 inhibits the production of several glycolytic intermediates and therefore prevents differentiation of T cell subtypes. On the contrary, TCR ligation and co-stimulator factors (CD28, ICOS) of Tfh potently activate glycolysis through PI3K-linked activation of mTOR signalling. In contrast, the generation of lipids promotes expression of C-X-C motif chemokine receptor 5 on the cell surface. Thus, nutrient metabolism and growth factor signalling are highly integrated processes to regulate Tfh cell immunity. For chronic inflammatory bowel disease (IBD), Wei et al. reported that G protein-coupled receptors (GPR)174 expedited the pathogenesis of IBD by regulating the immunologic crosstalk between dendritic cells (DCs) and T cells. GPR174 is widely expressed in most immune cells including T/B cells and DCs. Deletion of GPR174 in DCs impaired the function of activating naive CD4^+^T cells and instead induced more tolerogenic regulatory T cells.

Zn is an important and abundant trace mineral in human body. Sergi introduced an increased interest in supplementation of Zn which is possibly beneficial for immune function. There was a remarkable accumulation of naive CD4^+^T cells and T-cell receptor-derived excision circles (TRECs) after zinc-supplementation. TRECs are the most accurate measurement for evaluating thymus function. However, further studies are required to clarify the effects of trace elements and supplementations on boosting immune function in both healthy individuals and patients.

## Concluding Remarks

In the setting of disease, both intrinsic and extrinsic metabolic activity contribute to T cell-mediated immunity. In this section, we mainly discuss the metabolic regulation of T cells themselves. In fact, it is also critical to define the metabolic adaptations of T cells in specific tissue or circulation environments. For example, in sepsis sustained immune stimulation is caused not only by pathogens but also by the release of damage-associated molecular patterns (DAMPs) which trigger a vicious cycle of persistent immune activation and dysfunction (4). In peripheral circulation, the hyper-inflammation networks associated with sepsis affect immune cell metabolism and function. Combing plasma proteomic-metabolomics data of patients, it found that impaired fatty acid transport and β-oxidation, gluconeogenesis, and citric acid cycle were associated with increased risk of sepsis-related mortality (5). It is reasonable to suggest circulation metabolites link to risk stratification of patients with sepsis, which could be crucial for identification of patients who are more likely to benefit from specific clinical interventions.

The articles in this Research Topic are just some examples of the exciting new foyers into metabolic regulation of T cell immunity. As the new technologies of system immunology (single cell omics, spatial transcriptomics, etc.) grow in the field, we are granted clearer visons of how immune cells protect against pathogens and maintain tissue health.

## Author Contributions

All authors contributed to the article and approved the submitted version.

## Funding

This study was supported by the National Natural Science Foundation of China (No.81771672), Science and Technology Commission of Shanghai Municipality (20DZ2261200), and Special Fund for Clinical Research of Zhongshan Hospital, Fudan University (No.2020ZSLC07). The funding agencies had no role in the design and conduct of the study; in the collection, analysis and interpretation of the data; or in the preparation.

## Conflict of Interest

The authors declare that the research was conducted in the absence of any commercial or financial relationships that could be construed as a potential conflict of interest.

## Publisher’s Note

All claims expressed in this article are solely those of the authors and do not necessarily represent those of their affiliated organizations, or those of the publisher, the editors and the reviewers. Any product that may be evaluated in this article, or claim that may be made by its manufacturer, is not guaranteed or endorsed by the publisher.

## References

[1] Reina-Campos M Scharping NE Goldrath AW. CD8(+) T Cell Metabolism in Infection and Cancer. Nat Rev Immunol (2021) 21(11):718–38. doi: 10.1038/s41577-021-00537-8 PMC8806153 33981085

[2] Xu K Yin N Peng M Stamatiades EG Shyu A Li P. Glycolysis Fuels Phosphoinositide 3-Kinase Signaling to Bolster T Cell Immunity. Science (2021) 371(6527):405–10. doi: 10.1126/science.abb2683 PMC8380312 33479154

[3] McLane LM Abdel-Hakeem MS Wherry EJ. CD8 T Cell Exhaustion During Chronic Viral Infection and Cancer. Annu Rev Immunol (2019) 37:457–95. doi: 10.1146/annurev-immunol-041015-055318 30676822

[4] van der Poll T Shankar-Hari M Wiersinga WJ. The Immunology of Sepsis. Immunity (2021) 54(11):2450–64. doi: 10.1016/j.immuni.2021.10.012 34758337

[5] Langley RJ. An Integrated Clinico-Metabolomic Model Improves Prediction of Death in Sepsis. Sci Transl Med (2013) 5(195):195ra95. doi: 10.1126/scitranslmed.3005893 PMC3924586 23884467

